# A Role for Rebinding in Rapid and Reliable T Cell Responses to Antigen

**DOI:** 10.1371/journal.pcbi.1000578

**Published:** 2009-11-26

**Authors:** Omer Dushek, Raibatak Das, Daniel Coombs

**Affiliations:** 1 Department of Mathematics, University of British Columbia, Vancouver, British Columbia, Canada; 2 Institute of Applied Mathematics, University of British Columbia, Vancouver, British Columbia, Canada; 3 Department of Microbiology and Immunology, University of British Columbia, Vancouver, British Columbia, Canada; Imperial College London, United Kingdom

## Abstract

Experimental work has shown that T cells of the immune system rapidly and specifically respond to antigenic molecules presented on the surface of antigen-presenting-cells and are able to discriminate between potential stimuli based on the kinetic parameters of the T cell receptor-antigen bond. These antigenic molecules are presented among thousands of chemically similar endogenous peptides, raising the question of how T cells can reliably make a decision to respond to certain antigens but not others within minutes of encountering an antigen presenting cell. In this theoretical study, we investigate the role of localized rebinding between a T cell receptor and an antigen. We show that by allowing the signaling state of individual receptors to persist during brief unbinding events, T cells are able to discriminate antigens based on both their unbinding and rebinding rates. We demonstrate that T cell receptor coreceptors, but not receptor clustering, are important in promoting localized rebinding, and show that requiring rebinding for productive signaling reduces signals from a high concentration of endogenous pMHC. In developing our main results, we use a relatively simple model based on kinetic proofreading. However, we additionally show that all our results are recapitulated when we use a detailed T cell receptor signaling model. We discuss our results in the context of existing models and recent experimental work and propose new experiments to test our findings.

## Author Summary

T cells are essential players in the immune response to pathogens such as viruses and bacteria. They can be activated to respond when they recognize molecular signatures of infection (antigens) on the surface of antigen-presenting-cells of the immune system. The T cell response is highly specific (a particular T cell responds to only the right antigen), sensitive (a T cell will respond to as few as 1–10 antigens) and speedy (antigen binding may induce signaling within seconds). We wish to understand how the T cell, using its surface antigen receptors, is able perform this task. To do this, we developed mathematical models of antigens binding and unbinding from T cell surface receptors. Our primary finding is that T cells can discriminate antigens based on both their binding and unbinding rates from the T cell antigen receptor. We examine potential impacts of T cell antigen receptor clustering, T cell surface coreceptor molecules, and background self-antigens on this process.

## Introduction

T cells of the adaptive immune system use their T cell receptors (TCR) to scan the surfaces of antigen-presenting-cells (APC) for antigen in the form of specific peptides bound to major-histocompatibility complexes (pMHC). Scanning of APCs by T cells is rapid, with estimates suggesting that an individual T cell spends only 1–5 minutes interacting with a single APC if it lacks specific pMHC [1]. Experiments have demonstrated that T cells are extremely sensitive to specific pMHC, responding to as few as 1–10 pMHC in a sea of thousands of chemically similar self (null) pMHC [2],[3],[4],[5]. It has also been demonstrated that a single amino acid substitution on a presented peptide can dramatically alter the T cell response [6]. Speed, sensitivity, and specificity have been dubbed the S^3^ characteristics of antigen detection by T cells [1].

The observation that T cells transiently interact with APCs that do not express specific pMHC suggests that the decision to respond occurs within seconds of an encounter. Rapid turnover of T cell-APC contacts *in vivo* accelerates the search for specific pMHC by allowing numerous unique T cell-APC interactions. The decision to respond gives rise to a ‘stop’ signal [7] and is commonly followed by the formation of the immune synapse [8], a stable adhesion between the T cell and APC that persists for upwards of 30 minutes and facilitates a second, sustained phase of signaling.

Experiments and mathematical modeling have been extensively used to understand the efficiency of T cell activation. During the sustained signaling phase, the serial binding of many TCR by a single pMHC has been postulated to increase T cell sensitivity [9]. Serial binding is expected because the bonds formed between TCR and agonist pMHC are transient, with half-lives in the range of 1–100 s [10],[11],[12]. On the other hand, T cell specificity has been addressed by the *kinetic proofreading* model [13],[14]. This model postulates that a series of TCR-proximal steps, such as the binding and subsequent phosphorylation of the TCR associated immunoreceptor tyrosine-based activation motifs (ITAMs) by signaling molecules, occur upon pMHC binding, and that these signaling events require continued TCR engagement to proceed. A productive signal is transduced only after several such transformations have taken place. In this model, T cells are able to discriminate between different pMHC by imposing a threshold on the TCR-pMHC dissociation rate constant (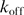).

Combining serial binding and kinetic proofreading reveals that a balance between sensitivity and specificity gives rise to an optimal 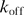 for efficient T cell activation [15],[16],[17], an effect which has been experimentally observed [18],[17],[19]. The efficiency of T cell activation in these models, and others [20],[21], is usually quantified by the number of activated TCRs (or the phosphorylation level of a downstream signaling molecule) integrated over the whole cell, and after a relatively long period of interaction (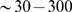 min) with an APC. Additionally, several studies have reported correlations between the TCR/pMHC bond dissociation constant (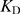), but not 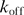, and the efficiency of T cell activation as measured after 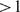 hour by cytoxicity and/or cytokine assays [12],[22],[23].

However, T cells have been observed to respond to stimulatory pMHC in less than a minute [1] and, at least for cytotoxic T cells, a stable contact interface is not required for pMHC detection [4]. Serial binding/kinetic proofreading models do not predict specificity on these short time scales, in part because signals generated by high concentrations of weakly binding self pMHC are found to be comparable to signals generated by low concentrations of high affinity agonist pMHC [16],[1]. Moreover, the early T cell response is unlikely to be determined by an equilibrium parameter, such as 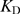, as it is quite unlikely that the T cell-APC interface attains equilibrium at such short times. It is more likely that 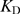 is an important determinant of the efficiency of T cell activation during the sustained phase of signaling, well after the initial decision to respond.

In this paper we investigate a putative mechanism for antigen discrimination during the early phase of TCR signaling. Specifically, we examine the role of TCR/pMHC rebinding in allowing T cells to make rapid (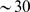 s) and reliable decisions to respond. By explicitly accounting for TCR/pMHC rebinding within existing formulations of diffusion-limited membrane reactions, we find that rebinding has very little effect in canonical proofreading models. A simple modification that accounts for signal persistence at the TCR allows individual TCR to integrate the duration of multiple rebinding events. The consequence of this scheme is that discrimination in this ‘sum-of-binding’ model is now sensitive to both the association and dissociation rate constants of the TCR-pMHC bond. This enhanced sensitivity leads to the finding that a T cell can discriminate between a wider spectrum of antigens than would be predicted by a traditional serial binding/kinetic proofreading model. We further show that coreceptors, but not TCR clustering, are important to achieve these rapid rebinding events. In addition, we show that signal persistence at the TCR does not allow high concentrations of endogenous pMHC to generate spurious signals. Finally, we show that our general conclusions are unchanged when our cartoon kinetic proofreading model is replaced by a detailed model of TCR-proximal signaling. We propose that T cells discriminate antigen based on 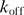 *and* 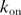 via a threshold in the sum-of-binding which allows for rapid and reliable T cell responses to specific pMHC.

## Results

### The effect of TCR/pMHC rebinding

**Figure 1:**
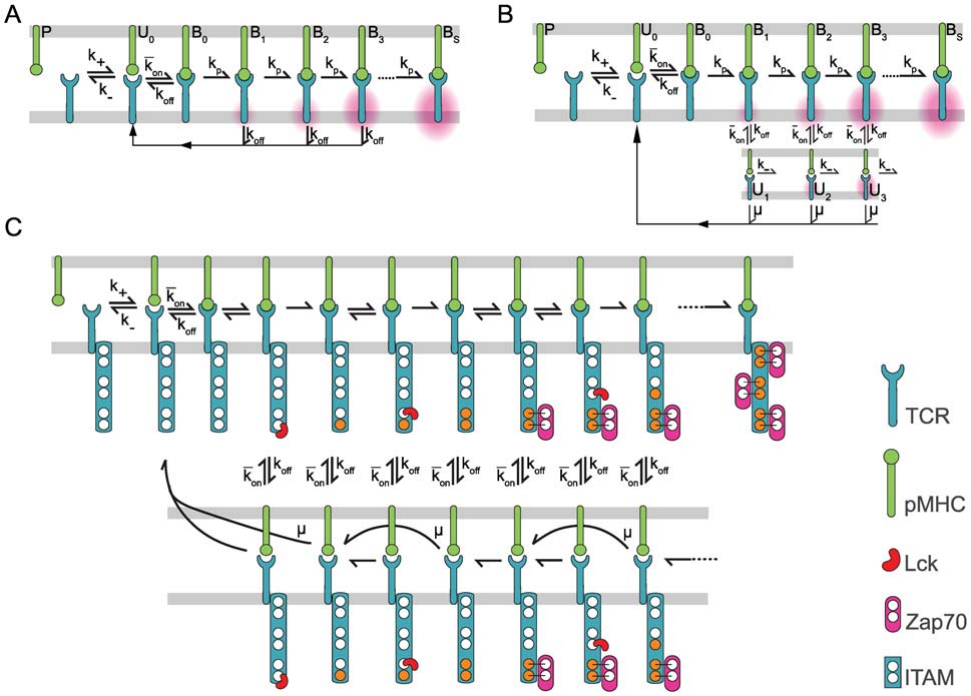
Rebinding in kinetic proofreading models. (A) Canonical kinetic proofreading postulates that the TCR proximal signaling events can be organized into successive steps that begin when pMHC binds TCR (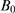). The TCR traverses through these steps (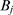) at a rate (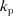) while the pMHC is bound and a productive signal is transduced only once a critical step has been reached (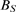). When unbound, the pMHC may diffuse away (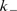) and subsequently bind another TCR (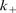). (B) Signal persistence allows the TCR to maintain its signaling state when pMHC unbinds (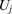). Three possibilities arise: (1) The pMHC may rebind the TCR which resumes proofreading (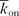), (2) The TCR decays to the unmodified state (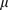), (3) The pMHC may diffuse away (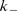). All rates are first order in units of 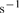 with the exception of 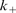 which is a second order rate in units of 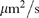. Main text results are focused on analyzing the generic models of panels (A) and (B) but our results are confirmed using a particular realization of TCR-proximal signals, shown in (C), which explicitly models the enzymatic activity of Lck in the sequential phosphorylation of a 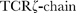 and the stabilization of a fully phosphorylated ITAM by Zap70. This model is adapted from Altan-Bonnet and Germain [40] and modified to include rebinding and signal persistence (bottom row). As in (A–B) when pMHC is unbound from TCR it may diffuse away (arrow not shown). In all three models the effective binding (coupling) rate (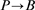) is 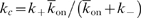 and the effective unbinding (uncoupling) rate (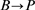) is 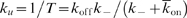. All models are described in the Methods.

We investigate the effect of rebinding between TCR and pMHC by modifying the canonical kinetic proofreading model [13] to explicitly account for the possibility of TCR/pMHC rebinding. The scheme is shown in Figure 1A, where the bound TCR goes through a series of 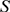 steps (e.g. binding and phosphorylation by signaling molecules), all occuring with an identical forward rate constant (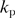), after which it becomes activated. We describe the system using a set of ordinary differential equations (ODE) where 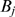 (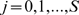) denotes the probability of the TCR being in the 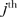 proofreading step. We assume that the pMHC is initially bound to TCR (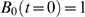, 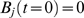 for all 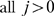). In addition to this modified kinetic proofreading model, we establish the role of rebinding in an explicit T cell signaling model (Figure 1C, discussed later).

The model explicitly accounts for a state where the pMHC is chemically dissociated but within physical proximity of the TCR (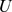) such that rebinding is possible. In this state, the pMHC can either re-bind the TCR (with a first order rate constant 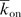) or diffuse away (also with a first order rate 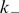). These first order rate constants (in units of 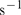) are related to the ensemble binding rate constant (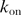) and the diffusion-limited association rate constant at the membrane (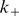) via 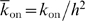 and 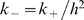, where 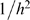 is the local TCR concentration (see Methods). In this model, the effective lifetime of a TCR/pMHC bond is: 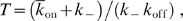
(1)the mean lifetime of an individual bond (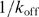) multiplied by the mean number of rebinding events (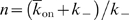). The number of rebinding events is then determined by the relative values of 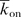 and 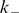. We note that the effect of rebinding described here is distinct from the serial binding of many different TCR by a single pMHC, which has been the topic of previous studies [24],[15],[25] and is usually implicitly captured by continuum mathematical models.

We verified the accuracy of our ODE model using spatial Monte Carlo simulations as described in the Methods. Briefly, we first established the accuracy of the simulation algorithm by comparing results to calculations from a partial-differential-equation (PDE) model that explicitly accounts for spatial effects. We next show that the spatial Monte Carlo simulations are in good agreement with the ODE model. Therefore, we conclude that this ODE model, which is computationally efficient to solve, accurately captures the effects of membrane diffusion.

**Figure 2:**
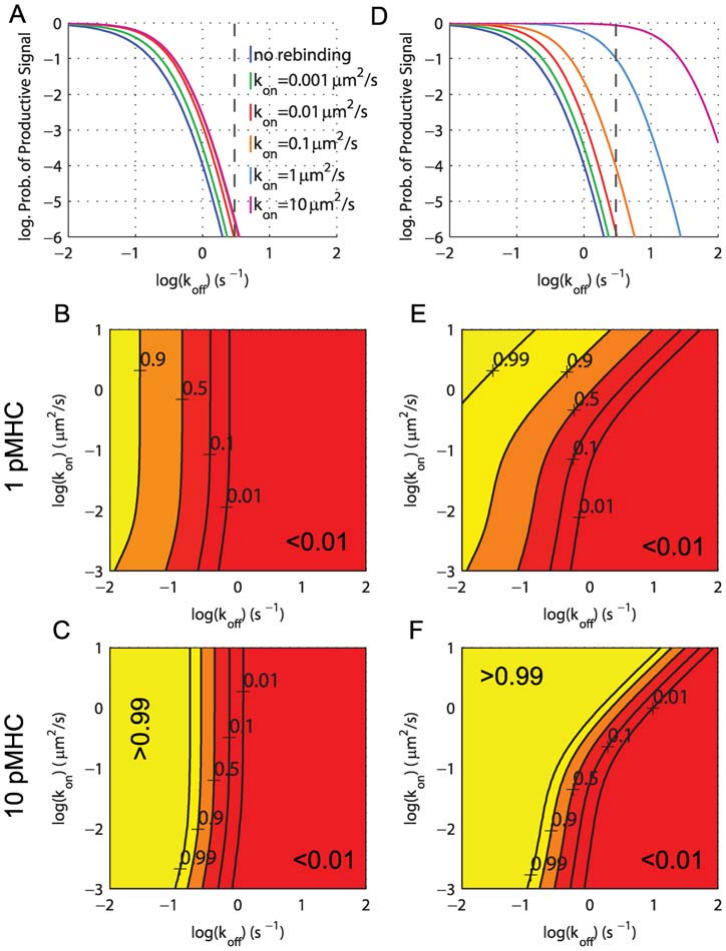
Productive signaling is determined by both 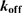 and 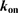 in a model of kinetic proofreading with signal persistence. Results are shown for kinetic proofreading (A–C) without signal persistence and (D–F) with signal persistence (see Figure 1). In all panels we show the probability of a productive signal (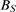) after 30 s and the threshold is set such that productive signaling requires 15 s of binding (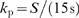, 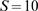). Shown in panels (A,D) is 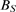 for indicated on-rates while panels (B,E) show contours of 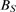 as a function of both 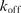 and 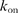 for a single pMHC. In panels (C,F) we show results for 10 pMHC by plotting the probability that at least 1 of 10 pMHC transduces a productive signal 
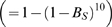
. Parameters: 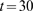 s, 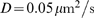, 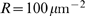 (TCR concentration), and 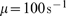.

To address antigen discrimination at short time scales, we chose a threshold of 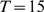 s (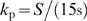) for a productive signal, and computed the probability of productive signaling after 30 s (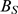) as a read-out for the T cell response. In Figure 2A we plot 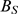 as a function of 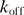 for a range of biologically relevant on-rates spanning two orders of magnitude in 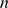. As expected, we observe a sharp productive signaling threshold in terms of 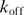. However, we find that accounting for rebinding events has only a small effect on antigen discrimination. Figure 2B shows contours of 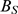 as a function of both 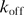 and 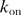 in the case of 1 pMHC (as in panel A) and in Figure 2C we show results when 10 pMHC are presented by plotting contours of the probability that at least 1 out of 10 pMHC elicits a productive TCR signal in 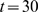 s. It is clear from the vertical contours that increasing the number of rebinding events (by increasing 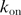) has only a negligible effect on productive signals in the canonical kinetic proofreading model.

### Signal persistence allows for 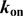 discrimination

We now examine a model within which TCR signaling is not disrupted by brief unbinding events, allowing a single TCR to integrate signals from multiple binding and rebinding events. This departure from a canonical proofreading model is plausible, given that a finite period of time is required to return the signaling state of the TCR to basal levels (e.g. by the action of phosphatases). We investigated this effect in our model, by introducing signal persistence states, denoted as 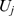, which represent unbound TCR from intermediate step 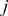 (Figure 1B). Unbound TCR in an intermediate step return to the unmodified state with rate 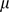 but kinetic proofreading resumes if the pMHC rebinds to the TCR. We chose a value of 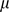 that allows for the signaling state of the TCR to persist for 0.01 s (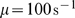). If 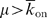 than signal persistence occurs rarely and when 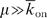 we recover standard kinetic proofreading. Results for several values of 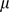 are discussed below in the context of endogenous pMHC.

As seen in Figure 2D, allowing for signal persistence leads to a much greater sensitivity to the on-rate. For example, for a pMHC with 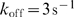 (dashed line in Figures 2D), we observe that an order of magnitude change in the on-rate increases the probability of productive signaling by several orders of magnitude. Comparing the contours of 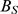 in the absence and presence of signal persistence (Figure 2; panels B vs. E and C vs. F) further illustrates this dependence on 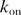 in the latter case. With signal persistence, the T cell is able to discriminate between pMHC based on both 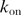 and 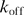 over a large portion of the parameter space. Examining Figure 2F, it is clear that in certain parameter regimes the T cell is still only able to discriminate pMHC based on 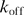. Discrimination is independent of 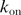 when 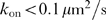 because for such small on-rates there is negligible rebinding before the pMHC diffuses away. In contrast to standard kinetic proofreading where the productive signaling threshold is determined entirely by 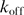 (Figure 2A–C), in our model the threshold depends on the total number of rebinding events, 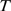 (Equation 1) and is therefore determined by both 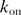 and 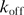. We refer to this model as **‘sum-of-binding’ discrimination**. Productive signaling can be achieved by a few binding events of long duration or by many binding events of shorter duration. We further support the notion of sum-of-binding discrimination in Text S1 by showing that this molecular model is comparable to a perfect detector that ‘samples’ and subsequently integrates the binding durations arising from a single pMHC, making the decision to respond based on a threshold in the sum duration of binding events.

### Potential effects of TCR clustering

Next, we investigated the role of TCR clustering in enhancing localized rebinding. Sub-micron TCR clusters have been observed immediately upon T cell contact with a supported planar bilayer containing specific pMHC [26],[27],[28], suggesting that they form within seconds. This rapid clustering is consistent with a simple diffusion-trapping mechanism, e.g. by cytoskeleton binding (see Text S2). Localized within these clusters are many signaling molecules important for T cell activation [29],[26],[27],[28] and there is strong evidence that coreceptors are present [30],[26]. After this rapid initial formation phase, TCR clusters translocate to the center of the contact interface forming a large scale aggregate. Their accumulation at the center, over a period of 5–10 minutes, is a marker for the formation of the immune synapse. The spontaneous formation of a few TCR clusters has also been observed in experiments using planar bilayers containing non-stimulatory (null) pMHC [28]. The function of TCR clustering remains controversial [31].

Given the observation that TCR proximal signals are confined to rapidly forming TCR clusters, we tested the possibility that TCR clustering can enhance detection of weakly binding pMHC. We utilized spatial Monte Carlo simulations to model a single pMHC interacting with TCR, and considered two different distributions of an identical number of TCR: 1) a homogeneous distribution at a concentration of 100 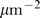 and 2) a specified number of TCR confined to a cluster of radius 0.1 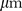 at the center of the domain surrounded by the remaining TCR distributed uniformly. To maximize the effect of clustering, we initialized the simulations with the pMHC bound at the center of a TCR cluster. All simulations were terminated when 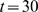 s or when a productive signal was attained. We take the simulation domain to be sufficiently large to avoid any boundary effects. The Monte Carlo simulation algorithm is described in the Methods.

**Figure 3:**
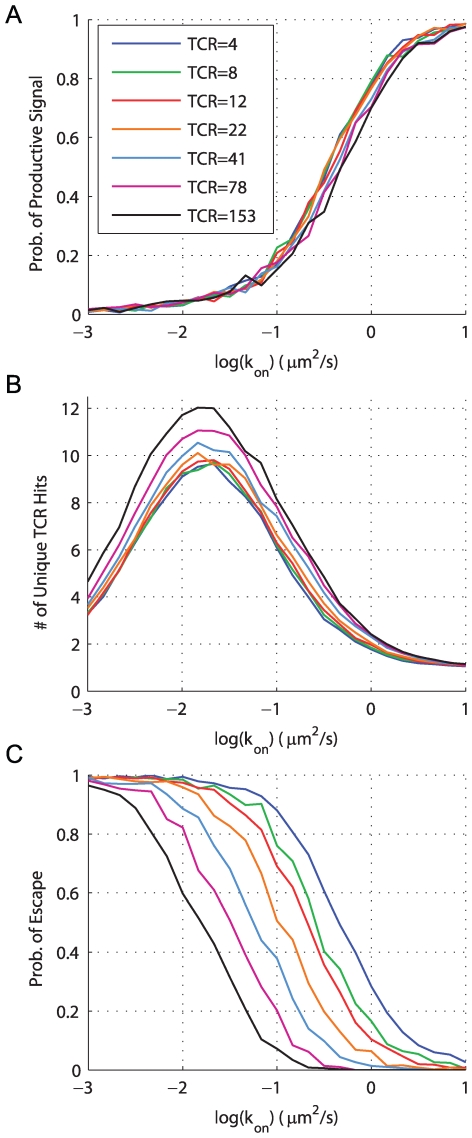
TCR clustering has no effect on productive signaling. We perform spatial Monte Carlo simulation of pMHC diffusing and reacting to TCR on a lattice. The simulation begins with the pMHC bound at the center of a TCR cluster (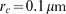) containing the indicated number of TCR and a homogeneous distribution of TCR (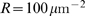) is assumed outside. Each TCR in the simulation is independent and performs stochastic kinetic proofreading with signal persistence. The simulation is terminated when 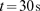 or a productive signal is transduced. (A) Fraction of simulations that are terminated by a TCR achieving productive signaling as a function of 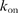 (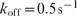) for several values of the number of TCR per cluster. (B) The number of unique TCR bound before the simulation terminates. A peak arises because rebinding to the same TCR is probable at large on-rates while at small on-rates serial binding of TCR is small. (C) Fraction of simulations that terminate with the pMHC outside of the TCR cluster. Each data point represents the mean of 500 simulations. Figure S1 shows results in the presence of coreceptors. Parameters: 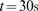, 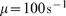, 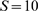, 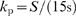, 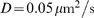.

In Figure 3A we plot the fraction of simulations that reached a productive signal in 30 s as a function of 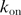 for the indicated number of TCR in the cluster. It is clear that increasing the number of TCR in the cluster has a negligible effect on productive signaling, though TCR clustering does increase the number of unique TCR bound by a pMHC (Figure 3B), and it substantially reduces the probability of escaping the cluster (Figure 3C). We conclude that receptor clustering has no impact on productive signaling, further underscoring the importance of pMHC rebinding to the same TCR versus serial binding of pMHC to different TCR.

### Increasing rebinding: potential effects of coreceptors

Another mechanism for increasing the number of rebinding events between a single pMHC and a single TCR is to include the T cell coreceptors CD4 or CD8 [32],[33]. Experiments have demonstrated that coreceptors bind MHC independent of TCR [34],[35]. The kinetics of TCR-coreceptor association are presently unknown, with some evidence suggesting constitutive association in resting T cells [36] and that TCR-coreceptor association increases on the time scale of minutes [37]. In our simple model, we assume that coreceptors are constitutively associated with TCR. In a following section, we use a detailed model of TCR-proximal signaling, in which coreceptors undergo reversible binding to TCR, and we show that our conclusions are unchanged.

**Figure 4:**
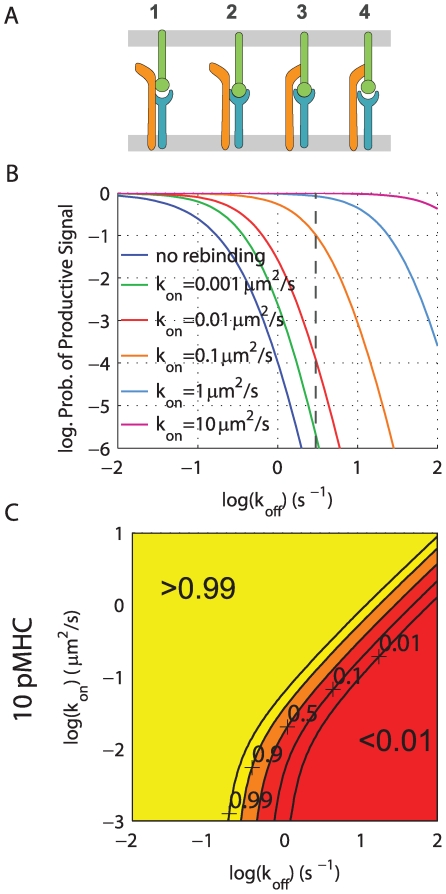
Coreceptors improve detection of weakly binding pMHC by promoting TCR/pMHC rebinding. (A) Coreceptors bind pMHC at a site that is independent of TCR and such a scheme is captured by a 4-state model. In this way, coreceptors decrease the effective mobility of pMHC allowing for rebinding to the same TCR. The probability of productive signaling in this case is shown in panels (B,C) which should be compared to panels (D,F) of Figure 2, respectively. We now find that the TCR/pMHC on-rate is an important determinant of productive signaling in almost the entire 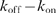 parameter space. Parameters: 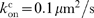, 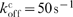, all other parameters as in Figure 2.

We used a heterodimerization model to capture the effect of coreceptors [38] (Figure 4A) and obtained binding parameters from the literature [34],[35],[36],[39]. The inclusion of coreceptors effectively decreases the mobility of pMHC, thus increasing the probability of rebinding TCR, and leading to a higher probability of productive signaling (Figure 4B vs. Figure 2D). We note that coreceptors increase sensitivity to weakly binding pMHC but do not enhance pMHC discrimination as such. Comparing Figure 4F to Figure 2C (no coreceptors), we conclude that, due to the presence of coreceptors, pMHC discrimination based on 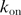 may occur over a wider range of parameter space. We also examine the role of coreceptors in the TCR cluster simulations described in the previous section. As expected, we find that coreceptors increase the probability of productive signaling but the clustering of TCR remains unimportant (Figure S1).

### Productive signaling by many endogenous pMHC is unlikely

**Figure 5:**
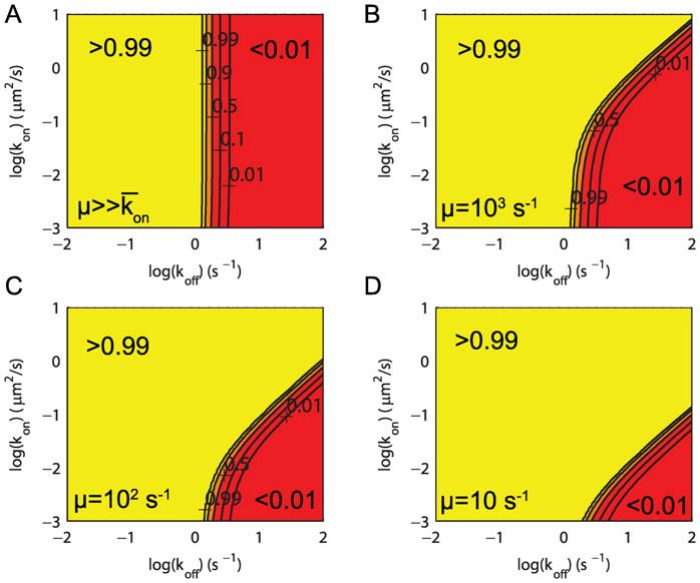
Productive signals by a high concentration of pMHC. Shown are contours of the probability that at least 1 TCR out of 7854 at the T cell - APC contact interface has transduced a productive signal when 39270 identical pMHC are presented on the APC with the indicated 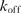 and 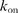. Shown are results (A) in the absence of signal persistence (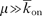), (B) with 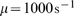, (C) 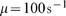 (used throughout this work), and (D) 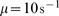. We see that despite a large number of pMHC, there is a substantial region of parameter space where self (null) pMHC (low 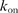, large 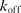) will be unable to transduce a productive signal through even a single TCR provided 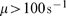. In the case of panel (D), we see that signal persistence at the TCR is sufficiently long to allow a sequence of different pMHC to activate a single TCR. Parameters: 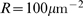 (TCR concentration), 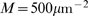 (pMHC concentration), interface radius = 5 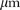, 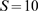, 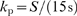, 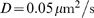, 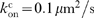, 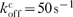.

A key advantage of signal persistence is that it allows the T cell to set a high threshold for productive signals (high specificity) while maintaining sensitivity to antigenic pMHC, provided they rebind. The threshold we have used is 15 s which requires pMHC to have 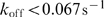 in order to frequently achieve productive signals by a single binding event (see Figure 4C for 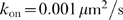). Thus, agonist pMHC with 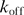 larger than this value will usually have to rebind (via a high 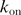) to achieve productive signals. The affinity and kinetics of endogenous pMHC binding to TCR have yet to be determined but are expected to be characterized by 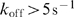 and endogenous pMHC are therefore unlikely to transduce productive signals in a single binding event. However, endogenous pMHC are present in large numbers and therefore sequential binding could result in activated TCR. We examine this possibility in our model by comparing the rate of signal decay at the TCR (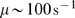) to a conservative estimate for the rate of endogenous pMHC binding to an individual TCR (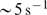, assuming 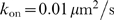 and that endogenous pMHC are present at a density of 500 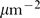). Intuitively, given the separation of scales between the binding and signal decay rates, we expect that the TCR will always revert back to the unmodified state before additional endogenous pMHC can bind. We confirm this physical argument by formulating the ODE model in the case of a high concentration of pMHC (see Text S3). In Figure 5 we show the probability that at least one TCR at the T cell-APC interface has achieved a productive signal when 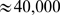 identical pMHC are presented. We find that unless 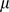 is very small (panel D), the region of parameter space corresponding to endogenous pMHC (large 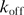, small 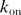) has a low probability of productive signals (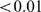).

### Rebinding in a detailed TCR-proximal signaling model

In the previous sections, we used generic proofreading models to test the role of localized rebinding and signal persistence. This raises the important question of whether our findings are relevant to more realistic TCR signaling models. To this end we have implemented a particular realization of TCR signaling based on the work of Altan-Bonnet and Germain [40]. The model is depicted in Figure 1C and represents a coupled system of 139 ODEs which were generated using BioNetGen [41] (see Text S4 for the BioNetGen file defining the model). The model explicitly accounts for the enzymatic kinetics of Lck sequentially phosphorylating the 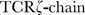 and stabilization of each ITAM by Zap70. A productive signal is defined as a fully phosphorylated 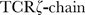 with three bound Zap70 molecules. We do not include further events, such as the phosphorylation of Zap70 which subsequently leads to the modification of cytosolic molecules involved in feedback (e.g. SHP-1, ERK). In the context of this model we are defining a productive signal, and hence a T cell response, as the generation of cytosolic molecules. As with the generic models we explicitly model rebinding and allow for signal persistence.

In Figure S2 we show the probability of a productive signal as a function of both 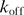 and 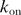 (A) without signal persistence, (B) with signal persistence, and (C) when including both signal persistence and coreceptors. We find that all our results are in qualitative agreement with those from the generic models presented earlier (Figure 2, 4), supporting the general applicability of our conclusions. Quantitative agreement is difficult to obtain because the threshold in the detailed model is determined by many individual parameters. Coreceptors are not constitutively associated with TCR in this model but undergo reversible binding. We have used a reasonable TCR-coreceptor affinity but if the TCR-coreceptor reaction on-rate is very small or the off-rate very large, coreceptors will have little impact on immobilizing pMHC.

## Discussion

We have investigated the role of TCR/pMHC rebinding in rapid T cell decisions to respond to specific pMHC. By allowing brief signal persistence, we have shown that a single TCR is able to integrate information from multiple pMHC rebinding events. In this model, the key determinant of specificity is a threshold in the sum-of-binding, T, which depends on both 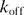 and 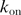 (equation 1). Strongly binding pMHC are able to overcome diffusive forces and rebind TCR while coreceptors are required to hold weakly binding pMHC during transient unbinding. Incorporating the spatial organization of TCR into a cluster has no additional effect on antigen discrimination in the model but does affect the motion of pMHC (e.g. by trapping it within the cluster [25]).

### 

#### Importance of 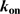


There are many factors that modulate initial T cell signaling at the T cell-APC interface, including receptor mobility, molecular concentrations, and the parameters governing the TCR/pMHC bond (e.g. 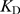, 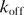, 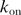). The majority of studies have focused on the dissociation rate constant, 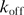, in determining T cell signaling. Here we have shown that the rate at which a pMHC rebinds to the same TCR may also be critical in determining T cell signaling.

Agonist pMHC with an off-rate that is too large to achieve a productive signal by a single binding event require multiple rebinding events. We have shown that coreceptors may effectively immobilize pMHC, allowing a single TCR to repeatedly ‘sample’ the pMHC. This result is based on an estimate of 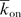 which is directly related to the 2D membrane on-rate (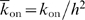), which itself is estimated from 3D SPR measurements (see Methods). Future experiments on the scale of a single TCR/pMHC are necessary to directly estimate 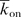. Experiments using stop-flow measurements in solution have provided evidence for a two-step binding scheme [42], whereby the second step is a factor of 10–100 slower than our estimate of 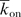. If such estimates are preserved on the T cell membrane, coreceptors may not be sufficient to immobilize pMHC to TCR and progressively higher order complexes (e.g pseudodimers [43]) will likely be important in allowing for TCR/pMHC confinement and rebinding [44]. Coreceptors may also be important in promoting signaling by localizing Lck to TCR [45].

#### Effect of TCR clustering

We have found that TCR clustering has no appreciable effect on productive signals in our model, suggesting that the observed clustering of TCR does not simply amplify signaling through increased local density of TCR. Although not important for productive signaling, the trapping of pMHC by a TCR cluster, which has been previously investigated [25], may be important to collect relevant pMHC at the center of the interface as was shown in B cells [46]. We note that the density of TCR witihn a cluster is presently unknown and if clustered TCR are very densely packed, the reaction radii of neighbouring TCR may overlap. In this case the chemical kinetics used in the model may no longer be parameterized by macroscopic 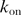 and 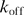 [47].

#### Model for initial T cell response

The present work motivates a model where pMHC binding to TCR for a sufficiently long time can trigger the formation of a TCR cluster and recruitment of coreceptors. Provided the signal to form a cluster is rapid enough, a simple diffusion-trapping model predicts that a TCR cluster can form in 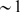 s and therefore, upon unbinding, the pMHC will be contained within a TCR cluster. In the context of our model, the formation of a TCR cluster and the exclusion of the membrane phosphatase CD45 [28], which can dephosphorylate the 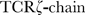 [48], will locally increase the signal persistence time and hence have a crucial role in limiting signal persistence to clustered TCR. Kinetic proofreading with signal persistence operating at the level of individual TCR in a cluster may then rapidly detect pMHC without the formation of a stable contact interface and therefore may underlie the decision to form the immune synapse.

#### Model validation

There are two key components of the model that need to be verified. The first is whether rebinding actually takes place between receptors and ligands at membrane interfaces. A high temporal-resolution FRET experiment where donor and acceptor are attached to receptor/ligand pairs could provide useful information on whether receptor and ligand rebind or move apart after chemical dissociation. Fluorescence recovery experiments at cell-bilayer interfaces can also be used to determine the rate at which cell surface receptors move apart from their ligands on the bilayer [49],[50]. Lower rates compared to surface plasmon resonance would suggest that rebinding may mediate longer receptor/ligand confinement, which has been reported for CD2-CD58 [49]. Additionally, single-particle-tracking of receptors in the presence and absence of their cognate ligand could provide the receptor/ligand confinement time at the single molecule level.

The second component that requires validation is whether rebinding has any functional consequences. This may be explored using a panel of pMHC variants that bind a particular TCR with various off- and on-rates. Examining T cell signaling shortly (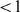 min) after each pMHC is displayed to the T cell may reveal the importance of 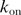. In particular, it will be important to show whether the early time response of each pMHC can be predicted based solely on 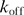 or 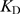, or if the sum-of-binding, 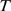, is the best descriptor of the early activity of pMHC. Note that 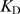 and 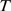 both depend on 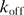 and 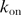 but exhibit a different functional form which allows them to be distinguished.

In the models we have described, the reaction on-rate governs the number of rebinding events between TCR and pMHC, and coreceptors act to increase this number by effectively reducing the lateral mobility of pMHC. Consistent with this interpretation, we find that a very small pMHC diffusion coefficient (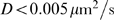 in contrast to measurements of 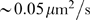 [51]) allow for pMHC detection in the absence of coreceptors (results not shown). In support of the present model, experiments have revealed that decreasing the lateral mobility of pMHC increases TCR signaling [52] and the sensitivity of T cells to antigen [53]. Therefore, a simple prediction of our model is that APC-presented pMHC that can be made non-stimulatory by blocking coreceptors should stimulate coreceptor-deficient T cells when immobilized on a surface.

Varma et al [28] observed the formation of a few transient TCR clusters in response to null (non-stimulatory) pMHC (Figure S2 D–E in their work). In our model, self (null) pMHC may bind many TCR in a cluster but in contrast to stimulatory pMHC, these molecules will be unable to rapidly rebind a single TCR. Therefore self pMHC may stochastically form a TCR cluster but individual TCR in the cluster serve as microscopic discrimination units exhibiting no response unless the sum of binding duration exceeds a threshold. A testable prediction of our model is that the number of spurious TCR clusters formed in response to null pMHC will increase as the concentration of null pMHC is increased. In this view, signals generated by self pMHC binding to TCR across the entire contact interface may be substantial but the decision to respond relies on a sequence of rebinding events, a feat that self pMHC are unlikely to perform. Self pMHC may be important during the sustained signaling phase on the timescale of tens of minutes [54],[55].

#### Relationship to existing models

Over the last several years it has become clear that antigen discrimination occurs over multiple space and time scales. Studies have provided evidence that discrimination occurs at the level of TCR (focus of present study), at the intracellular signaling level, and at the level of the T cell population [56]. Mathematical models and experiments focusing on TCR-proximal events on long time scales have revealed that an optimal bond lifetime is required for efficient T cell activation [18],[15],[16],[17], in apparent conflict with our prediction that pMHC activity monotonically increases as a function of the TCR-pMHC bond lifetime. A plausible reconciliation is that our model provides a basic threshold for the rapid initial discrimination of agonist pMHC, while other mechanisms that we do not consider (e.g. the accumulation of productive signals) possibly refine and amplify the discrimination over longer time periods.

The kinetic-segregation (KS) model [57] posits that disturbing a delicate kinase-phosphatase balance (by molecular segregation of phosphatases due to ectodomain size) is important for pMHC detection and discrimination. A spatial Monte Carlo model utilizing kinetic proofreading has revealed that productive signaling is sensitive to the TCR confinement time in kinase rich domains, which increases upon pMHC binding [20]. In our model, increases in the sum-of-binding via TCR/pMHC rebinding will also increase the TCR confinement time and in this respect, the two models are consistent.

Feedbacks in intracellular signaling molecules have also been implicated in antigen discrimination. Recent work has shown that positive and negative feedbacks (mediated by the intracellular molecules ERK and SHP-1, respectively) are important in producing sharp 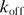 discrimination at 3–5 min [40],[58]. These intracellular molecules become modified by TCR that have completed several proofreading steps, and in turn supply feedback to modify the reaction rates governing proofreading at the TCR. Feedbacks involving the src-kinase Lck have also been implicated in antigen discrimination [59]. Feedbacks generate TCR hysteresis, as does signal persistence, with the important distinction that in our model rebinding allows the same TCR/pMHC pair to utilize the hysteresis, allowing rapid antigen discrimination based on 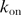. We have examined both general and specific models of TCR activation (Figure 1), to investigate the role of antigen rebinding and signal persistence. These effects are important for the very early T cell decision to respond, prior to the modification of cytosolic signaling molecules and the associated feedback mechanisms. Consequently, we have omitted feedback loops in our model. Further work is required to fully explore the effect of 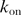 in mathematical models utilizing rebinding, feedback, and the longer time accumulation of productive signals.

Antigen discrimination may be considered as a series of gates whereby a specific pMHC must unlock each one for efficient T cell activation. These gates operate at various spatial scales (individual TCR, many intracellular molecules, etc) and over several time scales (e.g. initial T cell response vs. efficient T cell activation). We have focused on the very early time scales and on the smallest space scale (a single TCR) to show that the initial T cell response can be sensitive to both 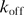 and 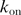 in a model that includes rebinding and signal persistence. The early time discrimination based on both 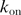 and 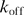 emerges, in part, due to explicitly modeling rebinding events, that have generally been ignored in previous mathematical models of T cell signaling. As we show, these effects are easily captured in ODE or PDE models by an additional compartment (as we have done here) or by altering the reaction rates to include the effect of diffusion (e.g. 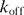 becomes an effective off-rate 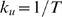 that depends on both 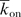 and diffusion in addition to 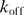, see Methods). We have proposed experiments needed to validate our model. We expect that, in future studies, these effects will be examined in models that operate on longer time scales and on multiple space scales, to establish a comprehensive picture of T cell activation.

## Methods

### Model for receptor-ligand interaction at membrane interfaces

We model the interaction between a single pMHC and a homogeneous TCR distribution at the T cell-APC interface using a two-step binding model [60],[61], 
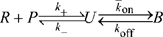
(2)where 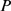, 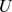, and 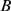 are the probabilities of finding the pMHC spatially separated from TCR (no binding may take place), unbound but within binding range of TCR, and bound to the TCR, respectively and 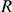 is the concentration of TCR. The diffusion-limited on-rate is given by 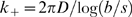, where 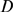 is the diffusion coefficient, 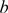 is the mean distance between TCR (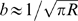), and 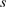 is the reaction radius of the TCR. When the pMHC is in state 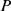, the rate at which it binds (or couples) TCR is 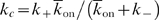 and when pMHC is in state 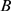, the rate at which it fully unbinds (or uncouples) from TCR is 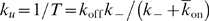. At equilibrium we expect that 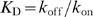 and therefore 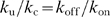. Assuming that 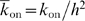, it then follows that 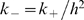. We define 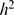 to be the area occupied by a single TCR and therefore 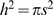. The parameter 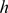 also sets the lattice size used in the spatial Monte Carlo simulations that are used to validate this ODE model (see below).

### Models for TCR-proximal signaling

We couple the above rebinding model to the three TCR signaling models shown in Figure 1. The detailed TCR-proximal signaling model is described in the main text and the two generic proofreading models (Figure 1A,B) are modeled with the following set of ODEs, 
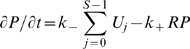


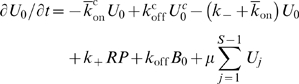


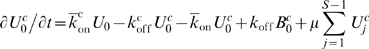


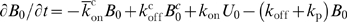


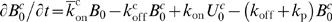


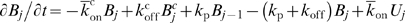


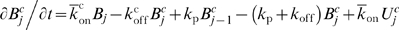


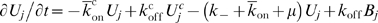


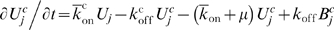


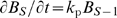


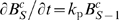
where 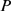, 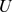 and 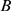 represent states where the pMHC and TCR are spatially separated, unbound but within binding proximity, and bound, respectively. We consider 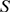 steps in the kinetic proofreading scheme with a forward rate 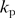 and denote intermediate states as 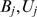. Coreceptor binding is denoted by a superscript 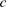. The pMHC may diffuse away (at rate 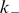) when it is unbound. We introduce signal persistence through the 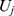 quantities which allow the TCR to remain in state 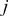 when the pMHC unbinds. In this state the unbound yet modified TCR may resume kinetic proofreading if the pMHC rebinds (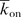), may return to the the unmodified state (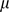), or the pMHC may diffuse away (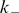). The reaction off-rate between TCR-pMHC is 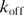 and between pMHC and coreceptor is 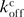. Since we are considering the interaction between a single pMHC and a single TCR the reaction on-rates are first order (in units of s^−1^) and can be related to macroscopic quantities by a reaction parameter (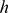): 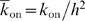 and 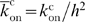. The macroscopic on-rates, 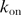 and 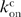, are 2D quantities in units of 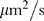 and can be related to experimentally determined 3D quantities, see below. Justification for the relations between the microscopic (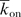) and macroscopic (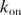) on-rate are provided below.

In all calculations the pMHC is initially bound to the TCR in state 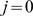 (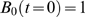, all other states are zero at 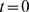). We set 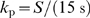 which corresponds to discrimination based on a threshold 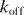 of 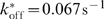 (canonical kinetic proofreading) and a threshold in the sum-of-binding (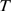) of 15 s (kinetic proofreading with signal persistence). In both cases the probability of a productive signal, after time 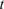, is 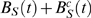. Numerical solutions are obtained using the Matlab function ode23.

### Validating the ODE model with spatial Monte Carlo simulations

We validate the ODE model described above with explicit spatial Monte Carlo simulations based on a discrete-space continuous-time model [62],[63]. We simulate a single pMHC diffusing on the APC membrane and binding to TCR on the T cell membrane. We use a lattice simulation which allows for reactions when the pMHC is in a lattice site opposite a TCR. The intrinsic binding rate is 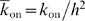, where 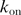 is the 2D (macroscopic) bimolecular reaction on-rate and 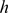 is the lattice spacing. The unbinding rate is 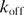 and diffusion (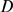) is captured by a first order reaction, of rate 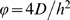, to nearest neighbour lattice sites. We take 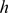 to be the size of a TCR (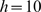 nm) in our simulations, making it possible to capture both reaction-limited and diffusion-limited regimes. Depending on the state of the pMHC only certain reactions are possible and the quantity 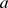 is the combined rate. In the absence of coreceptors, three states are possible: pMHC is unbound (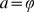), pMHC is unbound above TCR (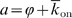), or pMHC is bound (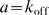). Based on the overall rate, 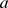, we compute the time for the next reaction, 
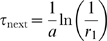
where 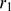 is a uniformly distributed random number between 0 and 1. Knowing 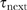, we use a second random number, 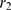, to determine which reaction actually took place. Finally, we simulate the reaction, advance time by 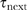, recompute 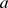, and repeat. For clarity we have described the method without kinetic proofreading, signal persistence, and coreceptors, but these effects just increase the number of possible reactions which we have implemented. This Monte Carlo algorithm is used to explore the effects of TCR clustering, as described in the main text.

We first tested the accuracy of our basic spatial simulations by comparing them to a deterministic partial differential equation (PDE) model. The simulations depend solely on intrinsic/microscopic quantities between individual TCR/pMHC (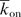, 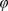) while the PDE model depends on ensemble/macroscopic quantities measured in bulk (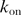, 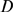). Simulations were initialized with the pMHC bound to TCR at the centre of the domain and terminated once the pMHC reached a distance 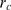. This termination conditions simulates a stochastic first passage time process. We performed 1000 simulations and in Figure S3 we show the binned (and normalized) first passage time (grey line). The PDE describing this first passage time process is, 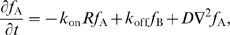


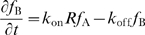
with 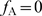 on the boundary (at 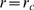), 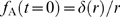, and 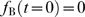. Note that 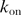 and 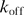 are the macroscopic quantities. To obtain the probability of a first passage we numerically solve the above PDE and compute the total flux through the boundary, 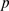, as a function of time. We plot 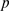 in Figure S3 (dotted black line) and find that it is in good agreement with the stochastic simulation in both the reaction- and diffusion-limited regimes. Good agreement is also observed over a wide range of parameters (not shown).

Having validated the Monte Carlo model with the PDE computations, we next implemented the complete Monte Carlo simulations that includes kinetic proofreading, signal persistence, and coreceptors and compared these simulations to the ODE model described above and used to generate Figures 2 and 4. As in the ODE computations, the simulations are now terminated after 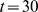 s or once a productive signal is transduced. In the first column of Figure S4 we show the fraction of simulations (out of 500 per data point) that terminated with a productive signal (colored circles) for A) kinetic proofreading, B) kinetic proofreading with signal persistence, and C) with the addition of coreceptors. We find good agreement with the ODE calculations (solid lines). In the second column we show good agreement across a wide range of TCR concentrations. We conclude that the ODE model accurately captures the effect of membrane diffusion.

### Model parameters

The calculations and simulations we have used rely on several parameters, many of which have been experimentally determined. The solution reaction parameters for many TCR/pMHC have been measured using SPR [10],[11],[12],[23]. Solution on-rates have been reported in the range of 500–300000 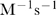 and off-rates in the range of 0.01–1 s^−1^. SPR measurements have revealed similar equilibrium binding constants between CD4 or CD8 and MHC [34],[35]. Wyer et al [34] report the solution on-rate to be 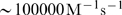 and the off-rate to be 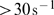. Experiments probing the interaction between CD8 and MHC in living cells report roughly similar on-rates [36] and off-rates [39]. We have used an off-rate of 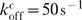 between MHC and coreceptors.

We have simulated reactions between membrane proteins and hence the bimolecular reaction parameter, 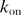, is a 2D quantity. However, reaction measurements between TCR-pMHC and CD4/CD8-MHC using SPR provide solution or 3D on-rates. To obtain estimates of 2D quantities we multiplied the 3D on-rate (in units of 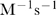) by a factor of 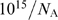 (where 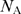 is Avagadro's number) to obtain units of 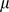
m^3^/s. We next divide this 3D quantity by a confinement length to obtain the 2D on-rate in units of 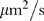. We use a confinement length of 0.262 nm, which can be obtained by comparing 3D and 2D dissociation constants [24],[64],[25]. This conversion indicates a range of 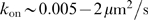 for TCR-pMHC on-rates and 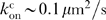 for CD4/CD8-MHC on-rates. Recent experiments have revealed membrane on-rates in this range for other T cell molecules [49]. Nonetheless, the accuracy of this method is unknown and we therefore explore a larger range in the reaction on-rates, see Figure 2 for example.

We have taken the diffusion coefficient of pMHC to be 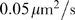 [51]. Calculations with 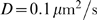 or 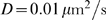 give similar results. We have assumed that the diffusion coefficient of TCR in a cluster is zero. We have assumed 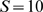 steps in the kinetic proofreading scheme but results are qualitatively similiar with any 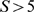. The rate of each step in the proofreading scheme, 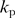, was equal and set to 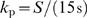. In order to obtain signal persistence the value of the decay-of-signaling parameter (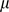) must be smaller than 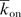. We have taken 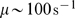 which may represent specific phosphatases having a concentration of 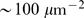 and effective on-rates of 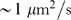. Standard kinetic proofreading models implicitly assume that 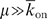 (i.e 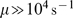).

## Supporting Information

Figure S1 Effect of including coreceptors in the TCR cluster simulations. We repeat all simulations as in Figure 3 except that we assume that coreceptors are associated with individual TCR in the cluster. Panels are analogous in both figures. (A) We find a general increase in the probability of productive signaling but TCR clustering still has no impact. (B) Number of unique TCR bound. (C) Fraction of simulations that terminated with the pMHC outside of the TCR cluster. Parameters: k^c^_on_ = 0.1 µm^2^/s, k^c^_off_ = 50 s^-1^ and all other values as in Figure 3.
(0.14 MB PDF)

Figure S2 Productive signaling in a detailed model of TCR-signaling and rebinding (Figure 1C). The model includes the sequential phosphorylation of the TCRζ-chain by Lck and the stabilization of doubly phosphorylated ITAM by Zap70. The model represents a system of 139 ODEs which are generated using BioNetGen and as before, the calculation runs from t = 0 s to t = 30 s with the pMHC initially bound to the TCR. A productive signal is defined as a fully phosphorylated TCRζ-chain bound by three Zap70 molecules. Results are shown as k_off_-k_on_ contour plots when 10 pMHC are presented (as in main text figures) in (A) the absence of signal persistence (μ = 10^12^ s^−1^), (B) the presence of signal persistence (μ = 100 s^−1^), and (C) in the presence of signal persistence and coreceptors. Comparisons to main text (Figure 2C, 2F, and 4C, respectively) reveals that generic kinetic proofreading accurately captures TCR-proximal signaling. In this model, coreceptors are not constitutively associated but reversibly bind TCR. The membrane concentration of coreceptors is taken at 100 µm^−2^ with an on-rate of 0.1 µm^2^/s and an off-rate of 10 s^−1^ . The effect of coreceptors (compare panel B to C) is lost if this TCR-coreceptor affinity is decreased by a factor of 10 (not shown). Parameters: All TCR/pMHC reaction-diffusion parameters are the same as in main text figures. The model includes additional parameters to describe the activity of Lck and Zap70 which we have taken from Altan-Bonnet and Germain [40]. The membrane concentration of Lck is taken to be 100 µm^−2^ , with an on-rate of 0.1 µm^2^/s, an off-rate of 30 s^−1^ , and a catalysis rate of 2 s^−1^. The cytosolic concentration of Zap70 is taken to be 2300 µm^−3^ with an on-rate of 0.02 µm^3^/s and an off-rate of 0.1 s^−1^.
(0.40 MB PDF)

Figure S3 Comparing the spatial Monte Carlo simulation to the relevant PDE computation of a reaction-diffusion first passage process. Simulations were performed in the (A) reaction-limited regime (k_on_ = 0.005 µm^2^/s) and (B) the diffusion-limited regime (k_on_ = 5 µm^2^/s). Parameters in the stochastic simulations are between individual proteins and were related to macroscopic/ensemble parameters used in the PDE model by k^-^_on_ = k_on_/h^2^ and ϕ = 4D/h^2^, where D and k_on_ are PDE parameters. We conclude that the spatial Monte Carlo simulation is accurate. Parameters: r_c_ = 1 µm, D = 0.05 µm^2^/s, k_off_ = 1 s^−1^, h = 0.01 µm.
(0.26 MB PDF)

Figure S4 Comparing the ODE calculations to a spatial Monte Carlo simulation. In all panels we show the probability of productive signaling for (A,D) kinetic proofreading, (B,E) kinetic proofreading with signal persistence, and (C,F) kinetic proofreading with signal persistence and coreceptors. Panels (A–C) are analogous to panels in the main text, showing productive signaling as a function of k_off_ for several values of k_on_. Panels (D–F) show results as a function of k_on_(k_off_ = 0.25 s^−1^) for several values of the TCR concentration. In all cases, we find good agreement between the spatial simulations (coloured circles) and the ODE calculations (solid lines). We conclude that the ODE model accurately captures the effect of membrane diffusion.
(0.45 MB PDF)

Text S1 Antigen discrimination by an idealized TCR
(0.10 MB PDF)

Text S2 Estimating the formation time of a TCR cluster
(0.02 MB PDF)

Text S3 Effects of endogenous pMHC
(0.02 MB PDF)

Text S4 BioNetGen code for the detailed TCR-proximal signaling model
(0.01 MB PDF)
