# Supplementary figures and images for: A Role for Rebinding in Rapid and Reliable T Cell Responses to Antigen
Source: PLoS Comput Biol. 2009 Nov 26;5(11):e1000578. doi: 10.1371/journal.pcbi.1000578 (PMC2775163; doi:10.1371/journal.pcbi.1000578)

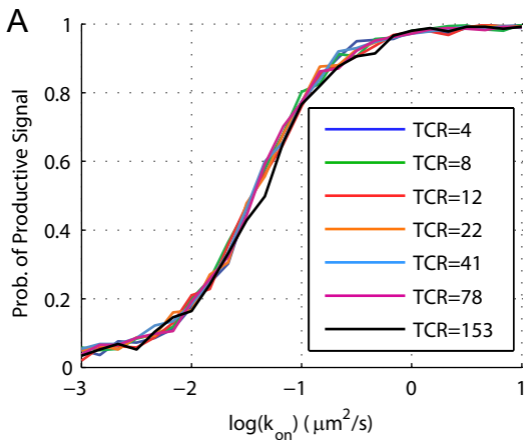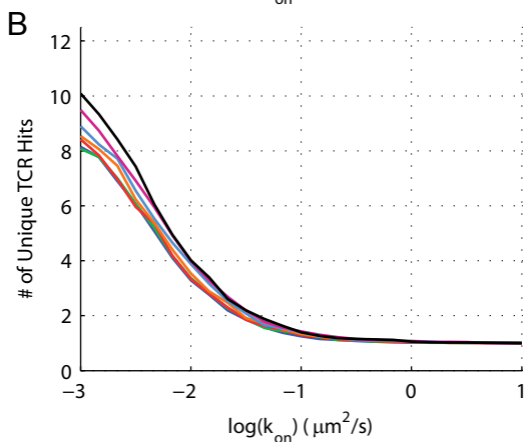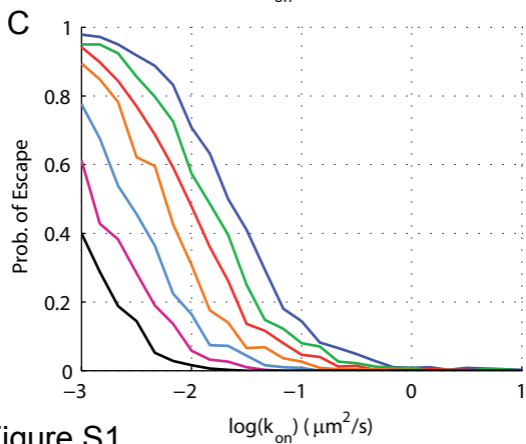

Figure S1

Supplement: Figure S1 — Effect of including coreceptors in the TCR cluster simulations. We repeat all simulations as in Figure 3 except that we assume that coreceptors are associated with individual TCR in the cluster. Panels are analogous in both figures. (A) We find a general increase in the probability of productive signaling but TCR clustering still has no impact. (B) Number of unique TCR bound. (C) Fraction of simulations that terminated with the pMHC outside of the TCR cluster. Parameters: kc on = 0.1 µm2/s, kc off = 50 s-1 and all other values as in Figure 3. (0.14 MB PDF) [file pcbi.1000578.s001.pdf]

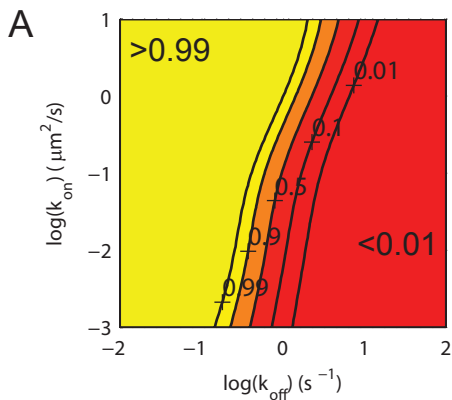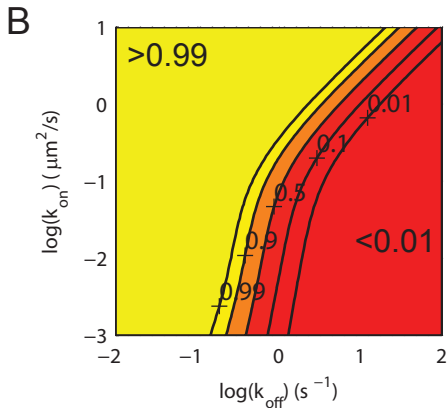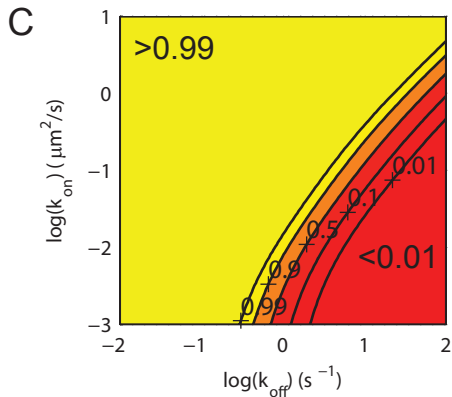

Figure S2

Supplement: Figure S2 — Productive signaling in a detailed model of TCR-signaling and rebinding (Figure 1C). The model includes the sequential phosphorylation of the TCRζ-chain by Lck and the stabilization of doubly phosphorylated ITAM by Zap70. The model represents a system of 139 ODEs which are generated using BioNetGen and as before, the calculation runs from t = 0 s to t = 30 s with the pMHC initially bound to the TCR. A productive signal is defined as a fully phosphorylated TCRζ-chain bound by three Zap70 molecules. Results are shown as koff-kon contour plots when 10 pMHC are presented (as in main text figures) in (A) the absence of signal persistence (μ = 1012 s−1), (B) the presence of signal persistence (μ = 100 s−1), and (C) in the presence of signal persistence and coreceptors. Comparisons to main text (Figure 2C, 2F, and 4C, respectively) reveals that generic kinetic proofreading accurately captures TCR-proximal signaling. In this model, coreceptors are not constitutively associated but reversibly bind TCR. The membrane concentration of coreceptors is taken at 100 µm−2 with an on-rate of 0.1 µm2/s and an off-rate of 10 s−1 . The effect of coreceptors (compare panel B to C) is lost if this TCR-coreceptor affinity is decreased by a factor of 10 (not shown). Parameters: All TCR/pMHC reaction-diffusion parameters are the same as in main text figures. The model includes additional parameters to describe the activity of Lck and Zap70 which we have taken from Altan-Bonnet and Germain [40]. The membrane concentration of Lck is taken to be 100 µm−2 , with an on-rate of 0.1 µm2/s, an off-rate of 30 s−1 , and a catalysis rate of 2 s−1. The cytosolic concentration of Zap70 is taken to be 2300 µm−3 with an on-rate of 0.02 µm3/s and an off-rate of 0.1 s−1. (0.40 MB PDF) [file pcbi.1000578.s002.pdf]

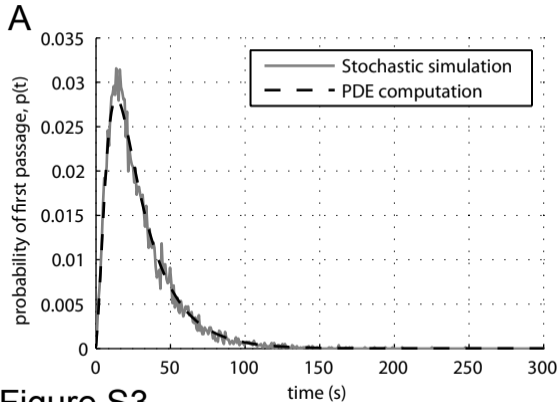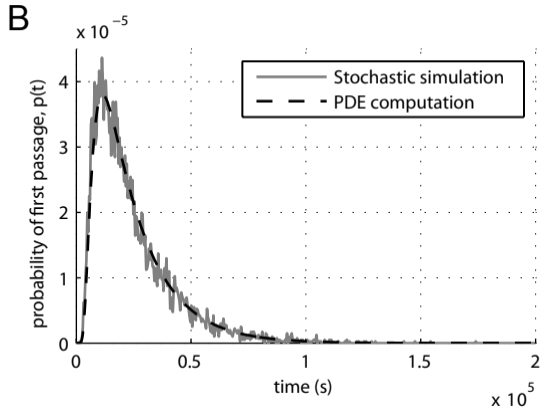

Figure S3

Supplement: Figure S3 — Comparing the spatial Monte Carlo simulation to the relevant PDE computation of a reaction-diffusion first passage process. Simulations were performed in the (A) reaction-limited regime (kon = 0.005 µm2/s) and (B) the diffusion-limited regime (kon = 5 µm2/s). Parameters in the stochastic simulations are between individual proteins and were related to macroscopic/ensemble parameters used in the PDE model by k- on = kon/h2 and ϕ = 4D/h2, where D and kon are PDE parameters. We conclude that the spatial Monte Carlo simulation is accurate. Parameters: rc = 1 µm, D = 0.05 µm2/s, koff = 1 s−1, h = 0.01 µm. (0.26 MB PDF) [file pcbi.1000578.s003.pdf]

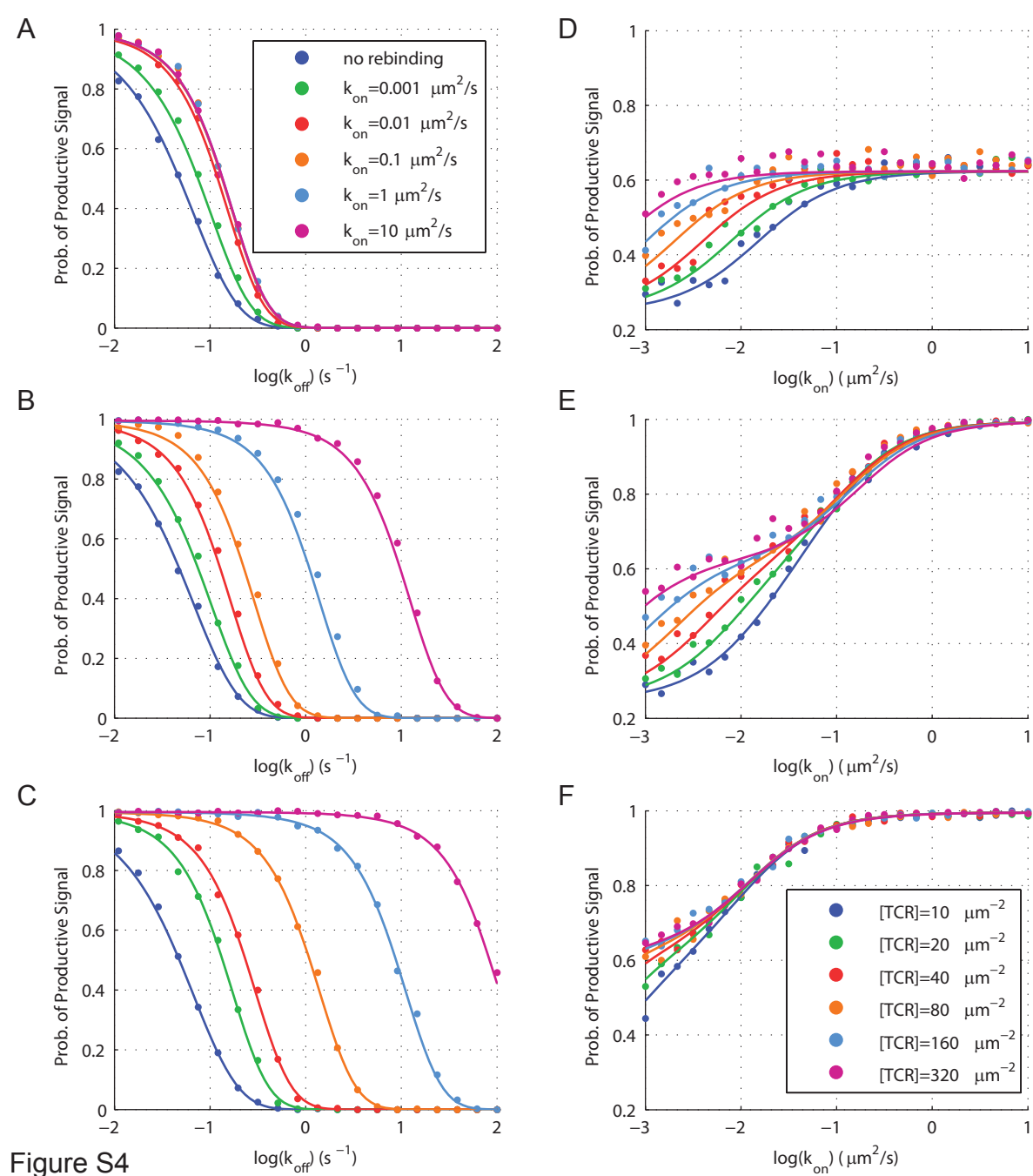

Figure S4

Supplement: Figure S4 — Comparing the ODE calculations to a spatial Monte Carlo simulation. In all panels we show the probability of productive signaling for (A,D) kinetic proofreading, (B,E) kinetic proofreading with signal persistence, and (C,F) kinetic proofreading with signal persistence and coreceptors. Panels (A–C) are analogous to panels in the main text, showing productive signaling as a function of koff for several values of kon. Panels (D–F) show results as a function of kon(koff = 0.25 s−1) for several values of the TCR concentration. In all cases, we find good agreement between the spatial simulations (coloured circles) and the ODE calculations (solid lines). We conclude that the ODE model accurately captures the effect of membrane diffusion. (0.45 MB PDF) [file pcbi.1000578.s004.pdf]
