# Supplementary material for: A Role for Rebinding in Rapid and Reliable T Cell Responses to Antigen
Source: PLoS Comput Biol. 2009 Nov 26;5(11):e1000578. doi: 10.1371/journal.pcbi.1000578 (PMC2775163; doi:10.1371/journal.pcbi.1000578)
Supplement: Text S1 — Antigen discrimination by an idealized TCR (0.10 MB PDF) [file pcbi.1000578.s005.pdf]

Text S1. Antigen discrimination by an idealized TCR

In the main text we have focused on the role of TCR/pMHC rebinding in molecular models of antigen discrimination and have shown that T cells are able to distinguish pMHC based on both k_{off} and k_{on}. In this text supplement we compare the molecular models of kinetic proofreading to calculations based solely on statistical distributions which represent an idealized TCR, limited only by stochasticity in the bond lifetime. We show that the molecular model of kinetic proofreading with signal persistence is comparable to an idealized TCR that 'samples' the binding durations arising from a single pMHC.

Serial binding can improve ligand discrimination based on the unbinding rate

In order to develop a full understanding of the discrimination problem faced by the T cell, we ask how well a perfect detector of binding can estimate k_{off} over multiple binding events. We imagine, therefore, a single pMHC that binds and unbinds from a single TCR, and we suppose that the T cell is able to perfectly measure the number and duration of binding events. In this highly idealized situation, how certain can the cell be that the actual k_{off} is below a threshold off-rate (k^*_{off})?

Since unbinding is an exponential stochastic process, the maximum likelihood estimate of k_{off} is the reciprocal of the mean binding time of observed binding events, $\hat{k}_{\text{off}} = n/\hat{T}_n$, where $\hat{T}_n = \sum_{i=1}^{n} \tau_i$ is the sum of binding durations from individual stochastic binding events (τ_i) [1]. To illustrate this, we let the threshold unbinding rate be $k^*_{\text{off}} = 1$ s^{-1} and simulate a series of n unbinding events for a particular k_{off}. In Figure P1A we plot the fraction of simulations (colored circles) where $\hat{k}_{\text{off}}$ is below k^*_{off}, as a function of the true off-rate (k_{off}) using different numbers of samples (n). This fraction is the statistical confidence which we can also calculate analytically as $P(\hat{k}_{\text{off}} < k^*_{\text{off}})$ using the distribution of T_n (solid lines). All simulations and calculations are described below. It is clear that discrimination improves as the number of binding events increase. Consider, for example, a self (null) pMHC with an off-rate that is three times larger than the critical off-rate ($k_{\text{off}} = 3$ s^{-1} here). We see that such self pMHC will be detected as antigenic at least 5% of the time based on a single sample but 0.0007% of the time based on 10 samples.

Sum-of-binding discrimination by an ideal detector

The unbinding rate detector described above relies on estimates of the sum of binding durations ($\hat{T}$) and the number of binding events (n) to compute $\hat{k}_{\text{off}}$. A simpler detector discriminates pMHC based on a threshold in the sum of binding durations. In this model the sum of binding durations ($\hat{T}$) must exceed a threshold (T^*) for productive signaling. As before, we simulate a series of n unbinding events and compute $\hat{T}_n$. In Figure P1B we plot the fraction of simulations where $\hat{T}_n > T^*$ as a function of the off-rate, using different numbers of binding events (n). Since there are multiple ways of obtaining T^* (few binding events of long duration or many binding events of short durations) the location of the threshold k_{off} is not constant and instead depends on n via $k^*_{\text{off}} = n/T^*$.

We note that both detectors shown in Figure P1 exhibit a favorable property, which is that the confidence is near 1 for pMHC having very small off-rates compared to the threshold after a single binding event ($n = 1$). In other words, a decision to respond based on a single binding event will be reliable for such

pMHC. This property is favorable because serial binding events from these pMHC could take more than 10 minutes. Serial binding events are most important for pMHC having an off-rate that is near the detection threshold.

Relation to kinetic proofreading

We compare the idealized detectors described above to molecular models in the lower panels of Figure P1. The on-rates shown in panels C and D correspond to $n = 1, 2, 5, 10, 25$ allowing for direct comparisons with the idealized TCR in panels A and B, respectively. Comparing canonical proofreading (Fig. P1C) with the idealized unbinding rate detector (Fig. P1A) we see that increasing the number of rebinding events shifts the curves to the right and therefore discrimination of whether $k_{\text{off}} < k^*_{\text{off}}$ becomes unreliable. For example, compare the two panels for pMHC having $k_{\text{off}} \sim 3$ s^{-1} and observe that multiple binding events *increase* the probability of signaling. This is simply because in contrast to the perfect detector, canonical proofreading treats each binding event as independent and therefore does not allow TCR to combine signaling from multiple binding events. We note that the results for this model (Fig. P1C) are similar to sum-of-binding discrimination (Fig. P1B) in the sense that the probability of productive signaling becomes larger for increasing on-rates (at a fixed off-rate).

Kinetic proofreading with signal persistence (Fig. P1D) qualitatively reproduces the perfect detector calculations for the sum-of-binding discrimination (Fig. P1B). Quantitative agreement is not possible because the perfect detector calculations are based on exactly n binding events while stochasticity in the model computations means that k_{on} only determines the mean number of binding events. The notion of an idealized detector was previously considered by Chan et al [2]. In their work, they focus on the likelihood of detection during a single TCR-pMHC binding event.

Method for calculating and simulating statistical confidence

We simulate a series of n unbinding events drawn from an exponential distribution with parameter λ using the Gillespie method [3],

$$\tau_i = \frac{1}{\lambda} \ln \left(\frac{1}{r} \right)$$

where r is a uniformly distributed random number on the interval (0,1) and τ_i is the binding duration. We define the sum of τ_i as $\hat{T} = \sum_{i=1}^{n} \tau_i$ and the maximum likelihood estimate of the true parameter is $\hat{\lambda} = n/\hat{T}$ [1]. For threshold detection of λ, we check if $\hat{\lambda} < \lambda^*$, where λ^* is the critical value required for detection. Repeating this simulation many times defines the probability that λ is less than λ^*, $P(\hat{\lambda} < \lambda^*)$. For threshold detection of T, we check if $\hat{T} > T^*$, where T^* is the threshold binding duration required for detection. Repeating this simulation many times defines the probability that T is greater than T^*, $P(\hat{T} > T^*)$. We use the terms probability and confidence interchangeably in this context.

We can obtain analytical expressions for the probabilities (P) by considering the probability density for T_n which is gamma distributed with parameters λ and n [1],

$$f(t|\lambda, n) = \frac{\lambda^n \exp(-\lambda t) t^{n-1}}{\Gamma(n)}$$

where Γ is the gamma function. Using the definition of $\hat{\lambda}$ we see that $P(\hat{\lambda} < \lambda^*) = P(T_n > n/\lambda_c)$, and therefore,

$$\begin{aligned} P(T_n > n/\lambda^*) &= 1 - P(T_n < n/\lambda^*) \\ &= 1 - \int_0^{n/\lambda^*} f(t|\lambda, n)\mathrm{dt} \\ &= 1 - \gamma(n\lambda/\lambda^*, n)/\Gamma(n) \end{aligned}$$

where γ is the lower incomplete gamma function. Summarizing we have,

$$\begin{aligned} P(\hat{\lambda} < \lambda^*) = & \quad 1 - \gamma(n\lambda/\lambda^*, n)/\Gamma(n) \\ P(\hat{T} > T^*) = & \quad 1 - \gamma(T^*\lambda, n)/\Gamma(n) \end{aligned}$$

In applying these results we have used $\lambda^* = k^*_{\mathrm{off}}$. We note that the probabilities only depend on relative values of λ (e.g. $P(\hat{\lambda} < \lambda^*)$ depends only on λ/λ^*).

References

1. Ross SM (2000) Introduction to Probability Models. Academic Press, 7th edition.

2. Chan C, George AJT, Stark J (2003) T cell sensitivity and specificity - kinetic proofreading revisited. Discrete and continuous dynamical systems 3:343–360.

3. Gillespie D (1977) Exact stochastic simulations of coupled chemical reactions. J Phys Chem 81:2340–2361.

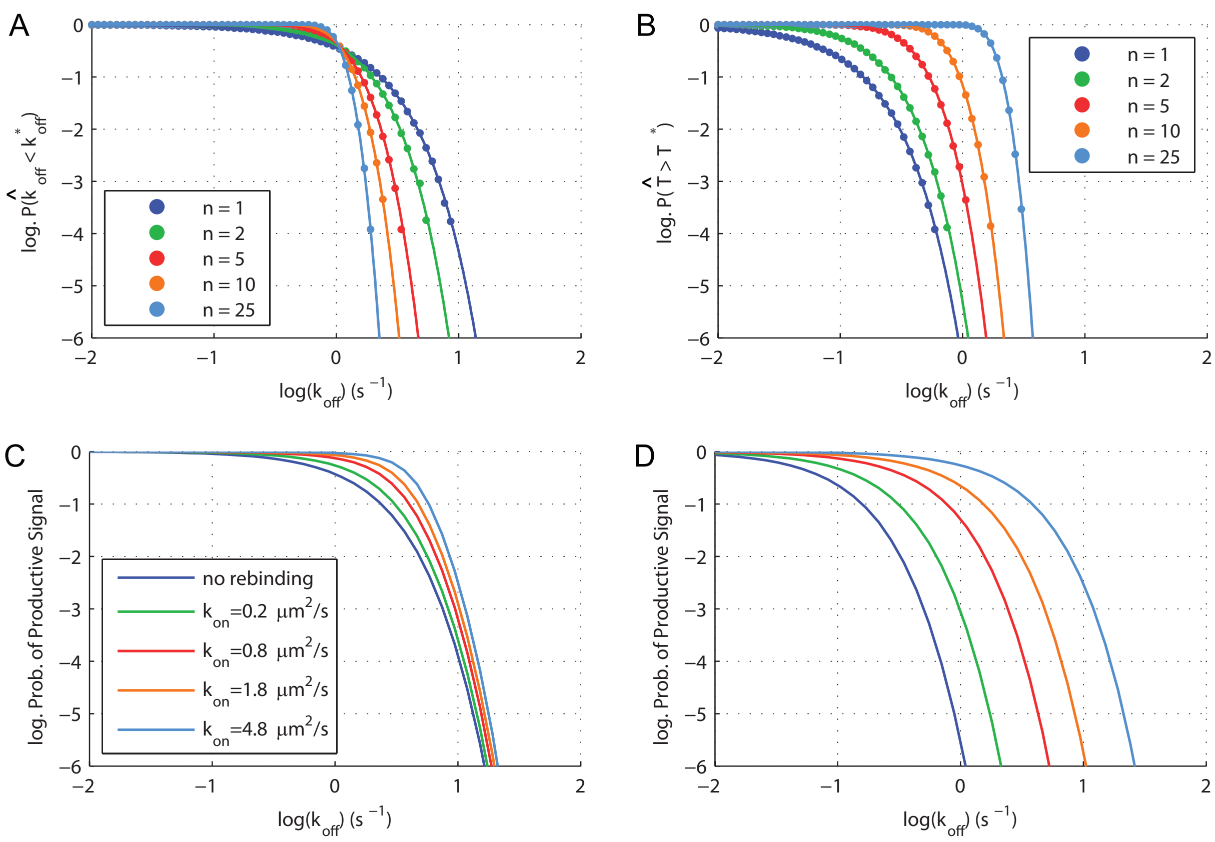

Figure P 1: Discrimination by (A,B) an idealized TCR and kinetic proofreading (C) without signal persistence and (D) with signal persistence. (A) Unbinding rate discrimination. Log confidence that $\hat{k}_{\text{off}}$ is below a threshold off-rate ($k^*_{\text{off}} = 1\ \text{s}^{-1}$) as a function of k_{off} for several values of n. (B) Sum-of-binding discrimination. Log confidence that the estimated sum of binding durations ($\hat{T}$) integrated across multiple binding events is greater than the threshold binding duration ($T^* = 15$ s). Note that the threshold in terms of k_{off} is not constant in (B) and depends on n by $k^*_{\text{off}} = n/T^*$. Mean of simulations (coloured circles) are in agreement with analytical calculations (solid curves). These calculations represent an idealized TCR that is able to precisely measure and integrate binding durations, limited only by the stochasticity in the TCR/pMHC bond. Molecular models are shown in the lower panels and computed using the models described in the main text (see Figure 1A-B). (C) Canonical kinetic proofreading is calibrated to detect $k^*_{\text{off}} = 1\ \text{s}^{-1}$ by taking $k_{\text{p}} = Sk^*_{\text{off}}$ and (D) kinetic proofreading with signal persistence is calibrated to detect $T^* = 15$ s by picking $k_{\text{p}} = S/(15\text{s})$. Parameters for molecular models as in Figure 2 in main text.
