# Supplementary material for: A Role for Rebinding in Rapid and Reliable T Cell Responses to Antigen
Source: PLoS Comput Biol. 2009 Nov 26;5(11):e1000578. doi: 10.1371/journal.pcbi.1000578 (PMC2775163; doi:10.1371/journal.pcbi.1000578)
Supplement: Text S2 — Estimating the formation time of a TCR cluster (0.02 MB PDF) [file pcbi.1000578.s006.pdf]

Text S2. Estimating the formation time of a TCR cluster

We can obtain an estimate for the time required to form a TCR cluster using the approximate theory of Weaver [1]. The model assumes a diffusion-mediated localization to a disc (cluster) on the surface of a sphere which is irreversible. The number of TCR in the cluster as a function of time follows

$$\text{TCR}(t) = RA_{\text{mc}} + RA_{\text{s}}(1 - \exp(-t/t_p))$$

where R is the background cell surface TCR concentration, A_{mc} is the TCR cluster area, A_{s} is the cell surface area, and t_p is given by

$$t_p = \frac{r_s^2}{D_T}\left[\frac{A_{\text{s}}/A_{\text{mc}}}{A_{\text{s}}/A_{\text{mc}} - 1}\ln(A_{\text{s}}/A_{\text{mc}}) - 1\right]$$

where r_s is the cell radius and D_T is the TCR diffusion coefficient. We obtain an estimate for the length of time required to localize $R_{\text{mc}}A_{\text{mc}}$ TCR by solving for t_{f} when $\text{TCR}(t_{\text{f}}) = R_{\text{mc}}A_{\text{mc}}$,

$$t_{\text{f}} = t_p \ln\left[\frac{RA_{\text{s}}}{RA_{\text{s}} + RA_{\text{mc}} - R_{\text{mc}}A_{\text{mc}}}\right]$$

where R_{mc} is the clustered TCR concentration. Using $r_s = 5\ \mu$m, $D_T = 0.06\ \mu\text{m}^2$/s [2], $A_{\text{s}} = 4\pi R_s^2$, $A_{\text{mc}} = 0.05\ \mu\text{m}^2$, $R = 100\ \mu\text{m}^{-2}$, and $R_{\text{mc}} = 500\ \mu\text{m}^{-2}$ [3] we approximate that a TCR cluster may form in $t_{\text{f}} \sim 1$ s. Decreasing the TCR cluster area, the final number of TCR in the cluster, or increasing the TCR diffusion coefficient further decreases the estimate of t_{f}.

References

1. Weaver DL (1983) Diffusion-mediated localization on membrane surfaces. Biophys J 41:81–86.

2. Dushek O, Mueller S, Soubies S, Depoil D, Caramalho I, et al. (2008) Effects of intracellular calcium and actin cytoskeleton on TCR mobility measured by fluorescence recovery. PLoS One 3:e3913.

3. Dushek O, Coombs D (2008) Analysis of serial engagement and peptide-MHC transport in T cell receptor microclusters. Biophys J 94:3447–3460.
