# Supplementary material for: A Role for Rebinding in Rapid and Reliable T Cell Responses to Antigen
Source: PLoS Comput Biol. 2009 Nov 26;5(11):e1000578. doi: 10.1371/journal.pcbi.1000578 (PMC2775163; doi:10.1371/journal.pcbi.1000578)
Supplement: Text S3 — Effects of endogenous pMHC (0.02 MB PDF) [file pcbi.1000578.s007.pdf]

Text S3. Effects of endogenous pMHC

We modify the ODE model presented in the main text to investigate the effects of a large concentration of identical pMHC molecules. In this calculation, we assume the contact interface to be a flat disc (radius 5 μm) containing a pMHC concentration of $M = 500\ \mu\text{m}^{-2}$ and a TCR concentration of $R = 100\ \mu\text{m}^{-2}$. We first reformulate the model from the perspective of a single TCR interacting with a homogeneous pMHC distribution. Next, we use this modified model to calculate the probability of productive signaling for a single TCR and assuming TCR do not compete for pMHC (i.e. M $\gg$ R), we calculate the probability that at least 1 TCR (out of 7854) at the T cell-APC contact interface has transduced a productive signal from interacting with 39270 pMHC.

In this model, we introduce additional states, denoted as $\hat{U}_j$, that track the probability of finding a TCR without pMHC (i.e. once it diffuses away) in an intermediate state. In this state, a different pMHC may bind TCR and resume signaling from where a previous pMHC left off (provided μ is sufficiently small). The ODE system describing this modified model is,

$$
\begin{aligned}
\partial B_0/\partial t &= -\overline{k}^{\text{c}}_{\text{on}} B_0 + k^{\text{c}}_{\text{off}} B^c_0 + k_{\text{on}} U_0 - (k_{\text{off}} + k_{\text{p}}) B_0 \\
\partial B^c_0/\partial t &= \overline{k}^{\text{c}}_{\text{on}} B_0 - k^{\text{c}}_{\text{off}} B^c_0 + k_{\text{on}} U^c_0 - (k_{\text{off}} + k_{\text{p}}) B^c_0 \\
\partial U_0/\partial t &= -\overline{k}^{\text{c}}_{\text{on}} U_0 + k^{\text{c}}_{\text{off}} U^c_0 - (k_- + \overline{k}_{\text{on}}) U_0 + k_+ M \hat{U}_0 + k_{\text{off}} B_0 + \mu \sum_{j=1}^{S-1} U_j \\
\partial U^c_0/\partial t &= \overline{k}^{\text{c}}_{\text{on}} U_0 - k^{\text{c}}_{\text{off}} U^c_0 - \overline{k}_{\text{on}} U^c_0 + k_{\text{off}} B^c_0 + \mu \sum_{j=1}^{S-1} U^c_j \\
\partial \hat{U}_0/\partial t &= k_- U_0 - k_+ M \hat{U}_0 + \mu \sum_{j=1}^{S-1} \hat{U}_j \\
\partial B_j/\partial t &= -\overline{k}^{\text{c}}_{\text{on}} B_j + k^{\text{c}}_{\text{off}} B^c_j + k_{\text{p}} B_{j-1} - (k_{\text{p}} + k_{\text{off}}) B_j + \overline{k}_{\text{on}} U_j \\
\partial B^c_j/\partial t &= \overline{k}^{\text{c}}_{\text{on}} B_j - k^{\text{c}}_{\text{off}} B^c_j + k_{\text{p}} B^c_{j-1} - (k_{\text{p}} + k_{\text{off}}) B^c_j + \overline{k}_{\text{on}} U^c_j \\
\partial U_j/\partial t &= -\overline{k}^{\text{c}}_{\text{on}} U_j + k^{\text{c}}_{\text{off}} U^c_j - (k_- + \overline{k}_{\text{on}} + \mu) U_j + k_+ M \hat{U}_j + k_{\text{off}} B_j \\
\partial U^c_j/\partial t &= \overline{k}^{\text{c}}_{\text{on}} U_j - k^{\text{c}}_{\text{off}} U^c_j - (\overline{k}_{\text{on}} + \mu) U^c_j + k_{\text{off}} B^c_j \\
\partial \hat{U}_j/\partial t &= k_- U_j - k_+ M \hat{U}_j - \mu \hat{U}_j \\
\partial B_S/\partial t &= k_{\text{p}} B_{S-1} \\
\partial B^c_S/\partial t &= k_{\text{p}} B^c_{S-1}
\end{aligned}
$$

where M is the pMHC concentration and all other parameters are defined in the main text. The probability of productive signaling through a single TCR is $B_S + B^c_S$ and the probability that at least 1 TCR at the T cell-APC contact interface has transduced a productive signal is simply $1 - (1 - B_S - B^c_S)^{7854}$. We plot this quantity in Figure 5 in the main text. As an example, consider an endogenous pMHC with $k_{\text{off}} = 5$ s^{-1} and $k_{\text{on}} = 0.001\ \mu\text{m}^{-2}$. When $\mu = 100\ \text{s}^{-1}$ this pMHC does not produce a productive signal (Figure 5C) despite forming $\sim 100,000$ TCR/pMHC bonds during 30 s ($= 1/(1/k_{\text{off}} + 1/(k_{\text{on}}[\text{pMHC}]))(7853$ $\text{TCR})(30\text{s}) = 107,100$).
