# Supplementary material for: A Role for Rebinding in Rapid and Reliable T Cell Responses to Antigen
Source: PLoS Comput Biol. 2009 Nov 26;5(11):e1000578. doi: 10.1371/journal.pcbi.1000578 (PMC2775163; doi:10.1371/journal.pcbi.1000578)
Supplement: Text S4 — BioNetGen code for the detailed TCR-proximal signaling model (0.01 MB PDF) [file pcbi.1000578.s008.pdf]

Text S4. BioNetGen code for the detailed TCR-proximal signaling model

```
#BioNetGen file for detailed model of TCR-proximal signals.

begin parameters

#Membrane concentrations (per um^2)
tLck    100
tCD4    100
tTCR    100

#Cytosolic concentrations (per um^3)
tZap70  2300

#We consider only a single pMHC (see text)
tpMHC    1

#Reaction parameter.
h=0.01

#TCR/pMHC encounter reactions (k+, k-)
kp  0.14*tTCR
km  0.14/(h*h)

#TCR/pMHC binding rates (both are extensively varied)
kp_on   0.1/(h*h)
kp_off  0.1

#Coreceptor/pMHC binding rates
kc_on    0.1/h^2
kc_off   50

#TCR/Coreceptor binding rates
kt_on   0.1
kt_off  10

#Lck-TCR binding/catalysis rates
Lkf 0.1
Lkb 30
Lkc 2

#Zap70-ITAM binding rates
Zkf 0.02
Zkb 0.1

#ITAM dephosphorylation rate
Fkc 100

#Consistency parameter
kmuf 1e12

end parameters

begin molecule types
Lck(b)
Zap70(b)
CD4(b)
TCR(b,U~0~1,P~0~1,C~0~1,ITAM1~U~P~2P,ITAM2~U~P~2P,ITAM3~U~P~2P,F~0~1~2)
end  molecule types

begin species
TCR(b,U~1,P~1,C~0,ITAM1~U,ITAM2~U,ITAM3~U,F~0) tpMHC
Lck(b)          tLck
Zap70(b)        tZap70
CD4(b)          tCD4
end species

begin reaction rules

#Formation of a TCR / pMHC encounter complex
TCR(U~0,P~0,C~0) <-> TCR(U~1,P~0,C~0)  kp,km

#TCR/pMHC binding
TCR(U~1,P~0) <-> TCR(U~1,P~1)  kp_on,kp_off

#Coreceptor/TCR binding
CD4(b) + TCR(b,C~0) <-> CD4(b!1).TCR(b!1,C~0)  kt_on,kt_off

#Coreceptor/pMHC binding
TCR(b!+,U~1,C~0) <-> TCR(b!+,U~1,C~1)  kc_on,kc_off

#ITAM-associated reactions

#Phosphorylation/Dephosphorylation + Zap70 stabilization of ITAM 1
    #ITAM1 + P
    Lck(b) + TCR(P~1,ITAM1~U,ITAM2~U,ITAM3~U,F~0) <-> Lck(b!1).TCR(P~1,ITAM1~U!1,ITAM2~U,ITAM3~U,F~1) Lkf,Lkb
    Lck(b!1).TCR(P~1,ITAM1~U!1,ITAM2~U,ITAM3~U,F~1) -> Lck(b) + TCR(P~1,ITAM1~P,ITAM2~U,ITAM3~U,F~1) Lkc
    #ITAM1 - P
```

```
    TCR(P~0,ITAM1~P,ITAM2~U,ITAM3~U,F~1) -> TCR(P~0,ITAM1~U,ITAM2~U,ITAM3~U,F~0) Fkc
    #ITAM1_P + P
    Lck(b) + TCR(P~1,ITAM1~P,ITAM2~U,ITAM3~U,F~1) <-> Lck(b!1).TCR(P~1,ITAM1~P!1,ITAM2~U,ITAM3~U,F~1) Lkf,Lkb
    Lck(b!1).TCR(P~1,ITAM1~P!1,ITAM2~U,ITAM3~U,F~1) -> Lck(b) + TCR(P~1,ITAM1~2P,ITAM2~U,ITAM3~U,F~1) Lkc
    #ITAM1_PP - P
    TCR(P~0,ITAM1~2P,ITAM2~U,ITAM3~U,F~1) -> TCR(P~0,ITAM1~P,ITAM2~U,ITAM3~U,F~1) Fkc
    #ITAM_PP + Zap70
    Zap70(b) + TCR(P~1,ITAM1~2P,ITAM2~U,ITAM3~U,F~1) <-> Zap70(b!1).TCR(P~1,ITAM1~2P!1,ITAM2~U,ITAM3~U,F~1) Zkf,Zkb
#Phosphorylation/Dephosphorylation + Zap70 stabilization of ITAM 2
    #ITAM2 + P
    Lck(b) + TCR(P~1,ITAM1~2P!+,ITAM2~U,ITAM3~U,F~1) <-> Lck(b!1).TCR(P~1,ITAM1~2P!+,ITAM2~U!1,ITAM3~U,F~1) Lkf,Lkb
    Lck(b!1).TCR(P~1,ITAM1~2P!+,ITAM2~U!1,ITAM3~U,F~1) -> Lck(b) + TCR(P~1,ITAM1~2P!+,ITAM2~P,ITAM3~U,F~1) Lkc
    #ITAM2 - P
    TCR(P~0,ITAM1~2P!+,ITAM2~P,ITAM3~U,F~1) -> TCR(P~0,ITAM1~2P!+,ITAM2~U,ITAM3~U,F~1) Fkc
    #ITAM2_P + P
    Lck(b) + TCR(P~1,ITAM1~2P!+,ITAM2~P,ITAM3~U,F~1) <-> Lck(b!1).TCR(P~1,ITAM1~2P!+,ITAM2~P!1,ITAM3~U,F~1) Lkf,Lkb
    Lck(b!1).TCR(P~1,ITAM1~2P!+,ITAM2~P!1,ITAM3~U,F~1) -> Lck(b) + TCR(P~1,ITAM1~2P!+,ITAM2~2P,ITAM3~U,F~1) Lkc
    #ITAM2_P - P
    TCR(P~0,ITAM1~2P!+,ITAM2~2P,ITAM3~U,F~1) -> TCR(P~0,ITAM1~2P!+,ITAM2~P,ITAM3~U,F~1) Fkc
    #ITAM2_PP + Zap70
    Zap70(b) + TCR(P~1,ITAM1~2P!+,ITAM2~2P,ITAM3~U,F~1) <-> Zap70(b!1).TCR(P~1,ITAM1~2P!+,ITAM2~2P!1,ITAM3~U,F~1) Zkf,Zkb
#Phosphorylation/Dephosphorylation + Zap70 stabilization of ITAM 3
    #ITAM3 + P
    Lck(b) + TCR(P~1,ITAM1~2P!+,ITAM2~2P!+,ITAM3~U,F~1) <-> Lck(b!1).TCR(P~1,ITAM1~2P!+,ITAM2~2P!+,ITAM3~U!1,F~1) Lkf,Lkb
    Lck(b!1).TCR(P~1,ITAM1~2P!+,ITAM2~2P!+,ITAM3~U!1,F~1) -> Lck(b) + TCR(P~1,ITAM1~2P!+,ITAM2~2P!+,ITAM3~P,F~1) Lkc
    #ITAM3 - P
    TCR(P~0,ITAM1~2P!+,ITAM2~2P!+,ITAM3~P,F~1) -> TCR(P~0,ITAM1~2P!+,ITAM2~2P!+,ITAM3~U,F~1) Fkc
    #ITAM3_P + P
    Lck(b) + TCR(P~1,ITAM1~2P!+,ITAM2~2P!+,ITAM3~P,F~1) <-> Lck(b!1).TCR(P~1,ITAM1~2P!+,ITAM2~2P!+,ITAM3~P!1,F~1) Lkf,Lkb
    Lck(b!1).TCR(P~1,ITAM1~2P!+,ITAM2~2P!+,ITAM3~P!1,F~1) -> Lck(b) + TCR(P~1,ITAM1~2P!+,ITAM2~2P!+,ITAM3~2P,F~1) Lkc
    #ITAM3_P - P
    TCR(P~0,ITAM1~2P!+,ITAM2~2P!+,ITAM3~2P,F~1) -> TCR(P~1,ITAM1~2P!+,ITAM2~2P!+,ITAM3~P,F~1) Fkc
    #ITAM3_PP + Zap70 (Irreversible, since downstream signaling, including feedback may begin here)
    Zap70(b) + TCR(P~1,ITAM1~2P!+,ITAM2~2P!+,ITAM3~2P,F~1) -> Zap70(b!1).TCR(P~1,ITAM1~2P!+,ITAM2~2P!+,ITAM3~2P!1,F~2) Zkf
#Ensure that all new pMHC binding events are to a 'fresh' TCR.
TCR(U~0,F~1) -> TCR(b,U~0,P~0,C~0,ITAM1~U,ITAM2~U,ITAM3~U,F~0)  kmuf
end reaction rules

begin observables
    PS TCR(F~2)
end observables

begin actions

generate_network({overwrite=>1});
simulate_ode({t_end=>1000,n_steps=>30});
writeMfile({});

end actions
```
